# The aneuploidy testing of blastocysts developing from 0PN and 1PN zygotes in conventional IVF through TE-biopsy PGT-A and minimally invasive PGT-A

**DOI:** 10.3389/frph.2022.966909

**Published:** 2022-09-05

**Authors:** Haijing Zhao, Ping Yuan, Xiaoli Chen, Haiyan Lin, Jun Zhao, Jia Huang, Qi Qiu, Xiaohui Ji, Qingxue Zhang, Wenjun Wang

**Affiliations:** Reproductive Medicine Center, Department of Obstetrics and Gynecology, Sun Yat-sen Memorial Hospital of Sun Yat-sen University, Guangzhou, China

**Keywords:** conventional IVF, 0PN/1PN blastocysts, aneuploidy, trophectoderm biopsy, miPGT-A

## Abstract

Zygotes without a pronuclear (0PN) or with one pronuclear (1PN) were defined as abnormal fertilization in conventional *in vitro* fertilization (IVF). The removal of 0PN and 1PN zygotes from conventional IVF cycles has always been controversial. This study aimed to investigate the chromosomal aneuploidy rates of 0PN- and 1PN-derived blastocysts in conventional IVF cycles and to assess the concordance rate between TE-biopsy PGT-A and miPGT-A. TE biopsies and culture media with blastocoel fluid (CM-BF) samples were whole-genome amplified by multiple annealing and looping-based amplification cycle-based single-cell ChromInst method. Next generation sequencing was performed for comprehensive chromosomal screening on a NextSeq550 sequencer using the NextSeq 500/550 High Output kit v2. The aneuploidy rates of 0PN-derived blastocysts were 19.7% for TE-biopsy PGT-A, and 36.1% for miPGT-A; the concordance rate for ploidy was 77.0%; and the sensitivity and specificity were 83.3% and 75.5%, respectively. The aneuploidy rates of 1PN-derived blastocysts were 37.5% and 37.5% by TE-biopsy PGT-A and miPGT-A, respectively; the concordance rate between TE biopsies and CM-BF samples was 83.3%; and the sensitivity and specificity were 77.8% and 86.7%, respectively. Regarding TE-biopsy PGT-A, there were no significant differences in aneuploidy rates among 0PN-, 1PN- and 2PN-derived blastocysts (PGT-M cycles) (19.7% vs. 37.5% vs. 24.3%, *P *= 0.226), but the aneuploidy rate of 1PN-derived blastocysts was slightly higher than the other two groups. An increase in aneuploidy rates was observed for 0PN/1PN-derived day 6 blastocysts compared to 0PN/1PN-derived day 5 blastocysts (TE-biopsy PGT-A: 35.7% vs. 19.3%, *P *= 0.099; miPGT-A: 39.3% vs. 35.1%, *P *= 0.705). The present study is the first that contributes to understanding the chromosomal aneuploidies in 0PN- and 1PN-derived blastocysts in conventional IVF cycles using TE-biopsy PGT-A and miPGT-A. The clinical application value of 0PN- and 1PN-derived blastocysts in conventional IVF should be assessed using TE-biopsy PGT-A or miPGT-A due to the existence of chromosomal aneuploidies.. In terms of consistency, the miPGT-A using blastocoel fluid enriched culture medium is promising as an alternative to TE-biopsy PGT-A for aneuploidy testing of 0PN- or 1PN-derived blastocysts in conventional IVF.

## Introduction

In conventional *in vitro* fertilization (IVF), the presence of two distinct pronuclear (2PN) and two polar bodies (pb) in zygotes 16–20 h after fertilization defines normal fertilization. Zygotes without a pronucleus (non-pronuclear, 0PN) or with one pronucleus (monopronuclear, 1PN) combined with the presence/absence of a second polar body are considered abnormally fertilized (1). To date, there is a general consensus that embryos derived from 1PN zygotes in intracytoplasmic sperm injection (ICSI) should be discarded for reproductive purposes to avoid a high proportion of aneuploids (2, 3). However, the removal of 0PN and 1PN zygotes from conventional IVF cycles has always been controversial. Most analyses suggested that blastocyst culture was a noninvasive choice for 0PN and 1PN embryo selection (4–6). Recently, a retrospective analysis revealed that 1PN-blastocyst transfers had a higher abortion rate and a lower live birth rate, and 0PN-blastocyst transfers had higher birth weights than 2PN blastocyst transfers (7), implying the role of genetic factors in 0PN and 1PN blastocysts. Although some cytogenetic analyses have indicated that half of the 1PN-derived embryos and 57%–62% of 0PN-derived embryos are diploid (8), there might be some limitations of fluorescence *in situ* hybridization (FISH) analyses. Recent array comparative genomic hybridization (aCGH) research showed that the aneuploidy rate of 1PN-derived blastocysts was 30.8% (4/13) in IVF and 33.3% (1/3) in ICSI, to 46.2% (6/13) in 2PN-derived blastocysts with IVF and 100% (3/3) in ICSI (9). Another study reported that the aneuploidy rates of 0PN-, 1PN-, and 2PN-derived blastocysts from conventional IVF and ICSI cycles were 24.4% (10/41) and 30.8% (8/26) and 38.2% (78/204) by aCGH or next generation sequencing (NGS), respectively (10). However, the sample size of the above studies was limited, and the aneuploidy rates of 0PN- and 1PN-derived blastocysts were below 2PN-derived blastocyst, consistent with other studies (1).

Preimplantation genetic testing for aneuploidy (PGT-A) using trophectoderm (TE) biopsy has been extensively applied as a diagnostic tool in assisted reproductive therapy because it improves pregnancy outcomes through comprehensive embryonic chromosome screening (11, 12). Nonetheless, the invasiveness, requirement of experienced technical skill, and the complex operational procedures of TE biopsies limit its widespread application (13). In addition, embryo mosaicism leads to false positives and false negatives in TE biopsy because the inner cell mass (ICM) cells are not tested (14). Furthermore, invasive biopsies may weaken the embryo developmental potential (15), and long-term biosafety of biopsies is yet to be assessed (16, 17). Noninvasive PGT-A with spent blastocyst medium (SBM) also has shortcomings, as SBM is associated with a higher maternal contamination (cumulus cells) than blastocoel fluid (BF), resulting in higher levels of nuclear and mitochondrial DNA being detected in the SBM, especially during the blastocyst stage (18, 19). Thus, a reliable, effective and minimally invasive strategy would promote the wider implementation of PGT-A. Embryonic cell-free DNA from blastocoel fluid (BF) (20–22) is an ideal genetic material for minimally invasive PGT-A (miPGT-A). After the necessary artificial shrinkage of blastocysts prior to vitrified cryopreservation, the junction of TE cells is broken by a laser pulse, or the aspiration of the injection pipette is used to release the BF into the culture medium (23, 24). The culture medium combined with BF is then collected for follow-up testing.

Currently, there are no full-scale investigations of chromosomes from 0PN/1PN-derived blastocysts in a conventional IVF by TE-biopsy PGT-A combined with miPGT-A available. Consequently, there are many unanswered questions. For example, is it necessary to detect aneuploidy in 0PN/1PN-derived blastocysts by TE-biopsy PGT-A or miPGT-A before the transfer? Is there any real aneuploidy rate difference between 0PN-, 1PN- and 2PN-derived blastocysts?

Therefore, for further clarifications, in this study, we investigated the chromosomal aneuploidy rates of blastocysts developing from 0PN or 1PN zygotes in conventional IVF cycles through TE-biopsy PGT-A and miPGT-A and we assessed the concordance of results from TE biopsies and culture media with blastocoel fluid (CM-BF), and compared the aneuploidy differences of 0PN-, 1PN- and 2PN-derived blastocysts.

## Materials and methods

### Study population

All patients were recruited between October 2019 and September 2020. A total of 101 blastocysts from 59 couples undergoing conventional IVF treatment were included in this study. Inclusion criteria: day 1: zygotes were 0PN with/without the second polar body or 1PN with/without the second polar body; day 3: 0PN- or 1PN-derived embryos with more than four cells were included; day 5 or day 6: the morphology score of blastocysts was above CC. Exclusion criteria: day 1: zygotes were 2PN, 3PN, and multi-PN; immature oocytes were excluded; day 3: 0PN- or 1PN-derived embryos with fewer than four cells were excluded; day 5 or day 6: embryos arrested in cleavage stage, or with morphology scores of CC were excluded.

**Figure 1:**
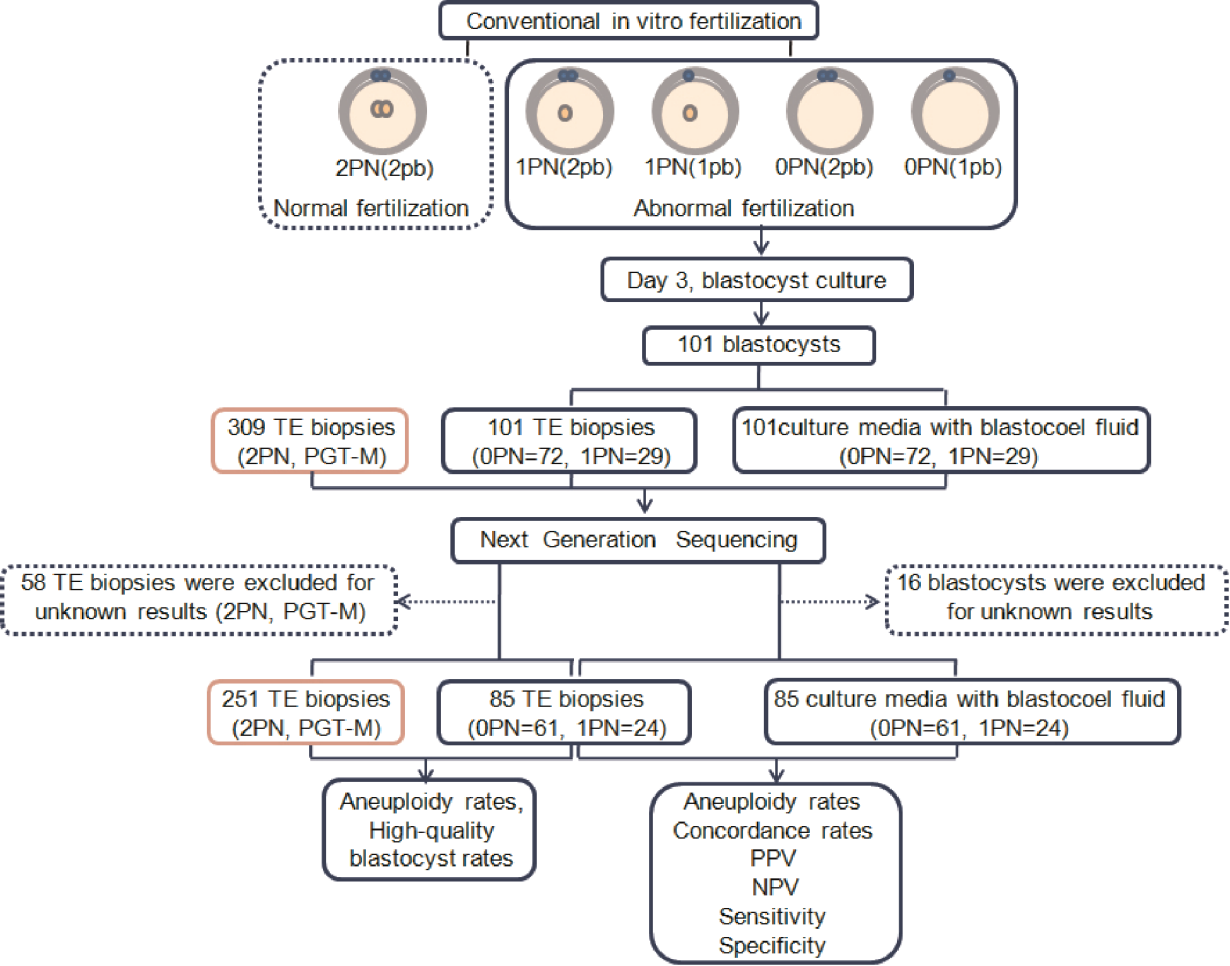
Flow diagram of the study. PN, pronuclear; pb, polar body; TE, trophectoderm; PGT-M, preimplantation genetic testing for monogenic diseases; PPV, positive predictive value; NPV, negative predictive value.

**Figure 2:**
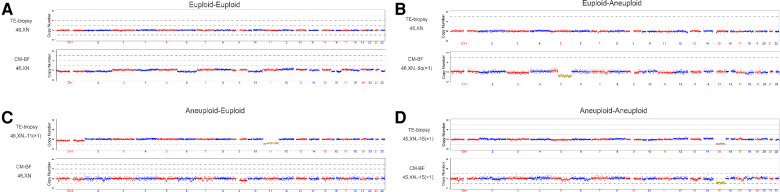
Examples of the comparison between TE-biopsy PGT-A and miPGT-A. (**A,D**) Concordance between TE biopsy and the corresponding CM-BF. (**B,**) Discordance between TE biopsy and the corresponding CM-BF. TE, trophectoderm; CM-BF, culture media with blastocoel fluid.

A flow diagram is shown (Figure 1). A total of 85 blastocysts (0PN = 61, 1PN = 24) with testing results from both TE and CM-BF samples were compared. The remaining 16 blastocysts without TE biopsies or CM-BF aneuploidy results were excluded. Four scenarios were considered when comparing the ploidy results of TE biopsies with the corresponding CM-BF: euploid-euploid, euploid-aneuploid, aneuploid-euploid, and aneuploid-aneuploid. The analysis of ploidy concordance included the matches euploid-euploid and aneuploid-aneuploid and for discordance euploid-aneuploid and vice versa (Figure 2). The analysis of total concordance included full concordance (all 24 chromosomes) and partial concordance (some concordant chromosomes). Similarly, the 2PN group (collection during the same period), including 309 TE biopsies from 77 PGT-M cycles, was recruited as the control group, and 58 TE biopsies were excluded for unknown PGT-A results, so 251 TE biopsies were compared to 61 TE biopsies in the 0PN group and 24 TE biopsies in the 1PN group.

### Ethics approval

This project was approved by the Institutional Medical Ethics Committee of Sun Yat-Sen Memorial Hospital. All participants signed an informed consent form after the study details were explained.

### Blastocyst culture

All 0PN, 1PN, and 2PN zygotes were cultured separately in G1-plus (supplemented with HSA; Vitrolife, Göteborg, Sweden) medium using alight paraffin oil overlay (Vitrolife, Göteborg, Sweden) on day one. 0PN-, 1PN- and 2PN-derived embryos (≥four cells) were individually placed in G2-plus (supplemented with HSA; Vitrolife, Göteborg, Sweden) medium using alight paraffin oil overlay (Vitrolife, Göteborg, Sweden) on day three, and they were further cultured till the blastocyst stage under 6% CO_2_, 5% O_2_, and balanced N_2_ at 37 °C.

Blastocyst morphology was evaluated using the Gardner scoring system (25). Blastocysts graded as 3–6, with either the ICM or TE graded other than C, were considered suitable for biopsy. High-quality blastocysts were scored as AA, AB, BA, or BB, and low-quality blastocysts were scored as BC or CB.

### Collection of CM-BF

A series of procedures have been implemented to avoid maternal cumulus cells contamination. 0PN- and 1PN-derived embryos of day 3 were washed several times to remove the loose cumulus cells on the zona pellucida before transferred to G2-plus culture medium. On day 5 or day 6, the original G2-plus droplet for blastocyst culture was discarded. The blastocyst was washed three times in different fresh droplets (pre-equilibrium) to fully remove the loose cumulus cells on the zona pellucida again. And then the blastocyst was transferred into another fresh 15 µl G2-plus droplet (pre-equilibrium) and perforated by a laser pulse (LYKOS®, Hamilton Thorne, INC., USA) for 200 µs to release the blastocoel fluid into the G2-plus droplet. After 4–6 h, the blastocyst was transferred into the biopsy dish, the mixture of G2-plus droplet with blastocoel fluid (15 µl) were collected and placed in an RNAse/DNAse-free PCR tube with 5 µl of phosphate-buffered saline (PBS) (Yikon, Jiangsu, China) (The collection method referred to the report of Jiao Jiao et al. (23).

**Figure 3:**
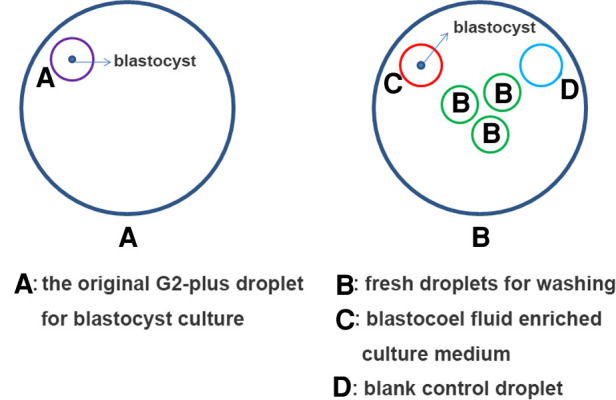
Schematic diagram of collecting blastocoel fluid enriched culture medium.

CM-BF samples were then marked clearly and immediately stored at −80 °C for subsequent testing. A 15 µl of G2-plus medium without embryo culture was used as a negative control (Figure 3).

### Collection of blastocyst TE cells

TE biopsy was performed on day 5 or day 6 by zona drilling with a laser, and a few biopsied TE cells (5–6 cells) were removed and collected for genetic analysis (26). Thereafter, TE cells were observed under a light microscope, and no adherent sperms were observed. Subsequently, TE cells were placed in RNAse/DNAse-free PCR tubes with 5 µl of PBS (Yikon Genomics, Jiangsu, China) and stored at −80 °C. Fresh PBS was used as a negative control. A single blastocyst was vitrified in sequence after biopsy, following the manufacturer's protocols (Kitazato Corporation).

### Whole-genome amplification (WGA) and NGS

WGA of biopsied TE cells and CM-BF samples was performed using multiple annealing and looping-based amplification cycle-based single-cell ChromInst method (Yikon Genomics, Jiangsu, China), according to the manufacturer's protocol. NGS used for comprehensive chromosomal screening was performed on a NextSeq550 sequencer (Illumina, USA) using the NextSeq 500/550High Output kit v2 (75 cycles) to generate >1 M valid reads of single-end 55 bp for analyzing the single-cell level chromosome copy number variation. The results were analyzed using Bioinformatics software ChromGo (http://chromgo.yikongenomics.cn:7000/#/home) (Yikon Genomics, Jiangsu, China) and aligned to the hg19 human reference genome (http://hgdownload.cse.ucsc.edu/downloads.html). The chromosomal aneuploidy and copy numbers of chromosomal segments larger than 4 Mb can be reliably predicted using the CBS algorithm (27).

### Pronuclear diameter measurement

All 1PN zygotes were photographed and their pronuclear diameters were measured by OCTAX Eye-WareTM Olympus system. Take the two largest diameters of the pronuclear plane as the vertical line, and take their mean as the pronuclear diameter (28).

### Judgment polar body number

Based on the size, shape and distance of two polar bodies, the judgment of polar body in 0PN or 1PN zygotes was as follows: 2PB: two integrated polar bodies can be clearly seen under the microscope, and there was a certain distance between them, or one integrated polar body and polar body fragments at a certain distance from it, or there was a certain distance between the two polar body fragments; 1PB: only one integrated polar body can be clearly seen under the microscope, or one integrated polar body and adjacent polar body fragments, or only one polar body fragments can be seen.

### Statistical analysis

All statistical analyses were performed using SPSS 22.0 (IBM). Female age is expressed as the mean ± standard deviation. A comparison of female age was performed using one-way ANOVA. The analysis is expressed as the percentage of probability. The chi-square test was used to compare the differences in percentages, and Fisher's exact test was applied in cases of low sample size. A logistic regression model was used to analyze the association between female age, polar body number, blastocyst score, pronuclear diameter, and aneuploidy rates of 0PN- and 1PN-derived blastocysts. The positive predictive value (PPV) and negative predictive value (NPV) were determined. PPV: true positive/(true positive + false positive) × 100. NPV: true negative/(true negative + false negative) × 100. Sensitivity and specificity were estimated for CM-BF related to TE biopsy as follows: Sensitivity: true positive/(true positive + false negative) × 100. Specificity: True negative/(true negative + false positive) × 100. All statistical tests were two-sided, and *P *< 0.05.

## Results

The aneuploidy rates of 0PN-derived blastocysts were 19.7% for TE-biopsy PGT-A, and 36.1% for miPGT-A; the concordance rate between TE biopsies and CM-BF samples was 77.0%; and the sensitivity and specificity were 83.3% and 75.5%, respectively. The aneuploidy rates of 1PN-derived blastocysts were 37.5% for TE-biopsy PGT-A, and 37.5% for miPGT-A; the concordance rate across TE biopsies and the corresponding CM-BF was 83.3%; and the sensitivity and specificity were 77.8% and 86.7%, respectively. The aneuploidy rates were high but not significantly higher in 1PN-derived blastocysts than in 0PN-derived blastocysts, regardless of the testing method used. There were no significant differences in the concordance rate, sensitivity, and specificity between the two groups (Table 1).

**Table 1:** Comparison of the results according to sample type and blastocyst type, % (*n*).

Characteristic	0PN-derived blastocysts	1PN-derived blastocysts	Total	*P* value
No. of blastocysts analyzed, *n*	72	29	101	–
blastocysts with informative TE + CM-BF	84.7% (61/72)	82.8% (24/29)	84.2% (85/101)	0.807
TE-aneuploidy rate	19.7% (12/61)	37.5% (9/24)	24.7% (21/85)	0.086
CM-BF-aneuploidy rate	36.1% (22/61)	37.5% (9/24)	36.5% (31/85)	0.902
PPV	45.5% (10/22)	77.8% (7/9)	54.8% (17/31)	0.132
NPV	94.9% (37/39)	86.7% (13/15)	92.6% (50/54)	0.652
Sensitivity	83.3% (10/12)	77.8% (7/9)	81.0% (17/21)	1.000
Specificity	75.5% (37/49)	86.7% (13/15)	78.1% (50/64)	0.577
Total concordance rate	77.0% (47/61)	83.3% (20/24)	78.8% (67/85)	0.731
Full concordance rate	37.7% (23/61)	41.65% (10/24)	38.8% (33/85)	0.736
Partial concordance rate	39.3% (24/61)	41.65% (10/24)	40.0% (34/85)	0.844

TE, trophectoderm; CM-BF, culture media with blastocoel fluid; PPV, positive predictive value; NPV, negative predictive value.

Total concordance includes blastocysts with full + partial concordances; full concordance is defined as TE and CM-BF both euploid or aneuploid for the same chromosomes, and partial concordance is defined as aneuploid blastocysts but with at least one identical aneuploid chromosome in TE and CM-BF; 0PN, zygote without pronuclear; 1PN, zygote with one pronuclear.

We compared the aneuploidy rates of 0PN- and 1PN-derived blastocysts in conventional IVF cycles and 2PN-derived blastocysts in PGT-M cycles. There were no significant differences in the TE-biopsies aneuploidy rates among 0PN-, 1PN- and 2PN-derived blastocysts (19.7% vs. 37.5% vs. 24.3%, *P *= 0.226), but the aneuploidy rate of 1PN-derived blastocysts was slightly increased compared to the other two groups. No significant differences were observed in the aneuploidy rates of day 5 blastocysts among the three groups and for day 6 blastocysts. However, in each group, the aneuploidy rate was slightly higher on day 6 than on day 5 (Table 2). Furthermore, the percentages of high-quality blastocysts were not significantly different among the three groups (60.7% vs. 54.2% vs. 52.6%, *P *= 0.526). However, the percentage of high-quality blastocyst on day 5 was significantly higher than those on day 6 in the 0PN group (71.7% vs. 26.7%, *P *= 0.005) and 2PN group (65.3% vs. 34.6%, *P *= 0.002 × 10^3^), as shown in Table 2.

**Table 2:** Comparison of the results from 0PN/1PN-derived blastocysts in conventional IVF and 2PN-derived blastocysts in PGT-M, % (*n*).

Characteristic	0PN-derived blastocysts	1PN-derived blastocysts	2PN-derived blastocysts	*P* value
Female age, years	31.5 ± 5.6	31.1 ± 4.0	31.7 ± 4.7	0.743
TE-aneuploidy rate	19.7% (12/61)	37.5% (9/24)	24.3% (61/251)	0.226
Day 5	17.4% (8/46)^a^	20.0% (2/10)^b^	21.1% (31/147)^c^	0.859
Day 6	26.7% (4/15)	50.0% (7/14)	28.8% (30/104)	0.280
High-quality blastocyst rate	60.7% (37/61)	54.2% (13/24)	52.6% (132/251)	0.526
Day 5	71.7% (33/46)^d^	70.0% (7/10)^e^	65.3% (96/147)^f^	0.705
Day 6	26.7% (4/15)	42.9% (6/14)	34.6% (36/104)	0.657

PN, pronuclear; TE, trophectoderm; IVF, *in vitro* fertilization; PGT-M, preimplantationgenetic testing for monogenic diseases.

^a^ TE-aneuploidy rate in 0PN-derived blastocysts: Day 5 vs. Day 6 (*P *= 0.681).

^b^ TE-aneuploidy rate in 1PN-derived blastocysts: Day 5 vs. Day 6 (*P *= 0.210).

^c^ TE-aneuploidy rate in 2PN-derived blastocysts: Day 5 vs. Day 6 (*P *= 0.158).

^d^ High-quality blastocyst rate in 0PN-derived blastocysts: Day 5 vs. Day 6 (*P *< 0.01).

^e^ High-quality blastocyst rate in 1PN-derived blastocysts: Day 5 vs. Day 6 (*P *= 0.240).

^f^ High-quality blastocyst rate in 2PN-derived blastocysts: Day 5 vs. Day 6 (*P *< 0.01).

For 0PN- and 1PN-derived blastocysts, the aneuploidy rates of day 5 blastocysts were 19.3% for TE-biopsy PGT-A, and 35.1% for miPGT-A; the concordance rate was 73.7%; and the sensitivity and specificity were 72.7% and 73.9%, respectively. The aneuploidy rates of day 6 blastocysts were 35.7% for TE-biopsy PGT-A, and 39.3% for miPGT-A; the concordance rate across TE biopsies and CM-BF samples was 89.3%; and the sensitivity and specificity were 90.0% and 88.9%, respectively. Regardless of the testing method, the aneuploidy rates of day 6 blastocysts were higher than those of day 5 blastocysts, without significant differences. There were no significant differences in the concordance rate, sensitivity, and specificity between the two groups (Table 3).

**Table 3:** Comparison of the results according to sample type and day of biopsy, % (*n*).

Characteristic	0PN/1PN-derived blastocysts		Total	*P* value
	Day 5	Day 6		
No. of blastocysts analyzed, *n*	69	32	101	–
blastocysts with informative TE + CM-BF	82.6% (57/69)	87.5% (28/32)	84.2% (85/101)	0.739
TE-aneuploidy rate	19.3% (11/57)	35.7% (10/28)	24.7% (21/85)	0.099
CM-BF-aneuploidy rate	35.1% (20/57)	39.3% (11/28)	36.5% (31/85)	0.705
PPV	40.0% (8/20)	81.8% (9/11)	54.8% (17/31)	0.057
NPV	91.9% (34/37)	94.1% (16/17)	92.6% (50/54)	1.000
Sensitivity	72.7% (8/11)	90.0% (9/10)	81.0% (17/21)	0.586
Specificity	73.9% (34/46)	88.9% (16/18)	78.1% (50/64)	0.334
Total concordance rate	73.7% (42/57)	89.3% (25/28)	78.8% (67/85)	0.170

PN, pronuclear; TE, trophectoderm; CM-BF, culture media with blastocoel fluid; PPV, positive predictive value; NPV, negative predictive value.

Total concordance includes blastocysts with full + partial concordances.

## Discussion

In this study, we analyzed the chromosomal aneuploidies in 0PN- and 1PN-derived blastocysts in conventional IVF cycles using TE-biopsy PGT-A and miPGT-A for the first time. It is found necessary that 0PN- and 1PN-derived blastocysts in conventional IVF should be advised by TE-biopsy PGT-A or miPGT-A due to the existence chromosomal aneuploidy.

Due to the debate on the availability of 0PN and 1PN zygotes in conventional IVF, the general consensus was to give 2PN zygotes, when they are available, priority over 0PN and 1PN zygotes, and the blastocyst culture would be applied to 0PN and 1PN zygotes. A retrospective cohort study revealed that the value of 1PN blastocyst FET would be without an increased risk of miscarriage rate, congenital malformations, or defects of psychomotor development similar to that of 2PN blastocyst FET (6). However, another study denied the above view and showed that 1PN blastocyst FET would increase miscarriage rate and decrease live birth rate compared with 2PN blastocyst FET (7). In our study, 1PN-derived blastocysts had a higher aneuploidy rate compared to 0PN- or 2PN-derived blastocysts by TE-biopsy PGT-A, which implied that 1PN-derived blastocysts had a higher risk of miscarriage. The aneuploidy rate of 0PN-derived blastocysts was the lowest among the three groups (Table 2), suggesting that transfer of 0PN-derived blastocysts did not seem to increase the risk of miscarriage and decrease the implantation rate, pregnancy rate and live birth rate (7, 29).

In addition, our study also revealed that day 5 blastocysts had lower aneuploidy rates and higher high-quality blastocyst rates than day 6 blastocysts, significantly in the 0PN and 2PN groups (Table 2). This implies that the single embryo should be transferred prior to day 5, regardless of it being in the 0PN, 1PN or 2PN groups, whereas day 6 blastocysts had lower pregnancy and live birth rates (30). Our in-house data on PGT also showed that day 5 single blastocyst FET had a higher clinical pregnancy rate (78.33% vs. 42.86%) and a lower miscarriage rate (4.26% vs. 20%) than day 6 (data not shown), which might be associated with the aneuploidy rate and embryo's developmental potential.

Furthermore, since the application of TE-biopsy PGT-A has been limited primarily due to its invasiveness and sampling bias with five to six cells extracted from the TE layer (31), the detection of chromosomal aneuploidy by noninvasive or minimally invasive methods should be considered. However, noninvasive PGT-A with SBM is also involved with maternal contamination (18, 19). Therefore, in our study minimally invasive PGT-A using BF was implemented, and laser-assisted collapse of the blastocoele cavity was used to release the BF. The consistency of TE biopsies and BF samples has been reported in other studies at lower rates (2.9% in PGT-M tests and 37.5% in PGT-A tests (18). Nevertheless, in our study, the concordance between TE-biopsies and CM-BF was high (78.8%) and reached 89.3% on day 6 blastocysts (Table 3). The highest concordance was observed in TE-biopsy PGT-A vs. miPGT-A. The concordance of 0PN- and 1PN-derived day 6 blastocysts between TE-biopsy PGT-A and miPGT-A was higher than of day 5 blastocysts (89.3% vs. 73.7%, *P *= 0.170), which was consistent with the report of Rubio et al. (84.0% vs. 63.0%, *P *= 0.029) (31). Moreover, in our study, when analyzing the discordance rates between TE biopsies and CM-BF samples, false positives were 21.9% (14 of 64), false negatives were 19.0% (4 of 21), and the corresponding sensitivity and specificity were 81.0% and 78.1%, respectively. Particularly on day 6, miPGT-A had a higher predicted value with increased sensitivity (90.0%) and specificity (88.9%) in comparison with previous studies where the sensitivity and specificity were 73.3% and 66.7% (32), 88.2%, and 84.0% (13), and 80.0% and 61.0% (33), respectively.

According to the formation mechanism of 0PN and 1PN zygotes described in previous reports (8, 34, 35), there might be three possible situations: (1) disappearance or delayed formation of female and male pronuclei during fertilization; (2) female pronucleus and male pronucleus fused and formed diploid 1PN zygotes; and (3) parthenogenetic activation. The FISH analysis demonstrated that 62.5% (10/16) of 1PN zygotes from IVF were haploid, suggesting that these 1PN zygotes might be due to parthenogenetic activation (35). Furthermore, based on the haplotype analysis using NGS, there were 40%–42.83% diploid biparental in 1PN-derived blastocysts from ICSI and 75.51% in 0PN-derived blastocysts and 80.13% 2PN-derived blastocysts, respectively (1, 36). However, there were no haplotyping analyses of 0PN or 1PN from conventional IVF. In our study, we did not exclude uniparental disomy and ploidy anomalies due to the limitation of TE-biopsy PGT-A, which requires further investigation.

In addition, other factors, including female age, polar body number, pronuclear diameter and blastocyst score, were also investigated in our study. Advanced maternal age contributes to an increased incidence of chromosomal aneuploidies (37). Our logistic regression showed that there were significant associations between female age, blastocyst score, and TE-biopsy aneuploidy rate; however, no significant association was observed between the polar body number and TE-biopsy aneuploidy rate in 0PN-derived blastocysts (Supplementary Table S1). The phenotype of blastocyst score associated with chromosomal aneuploidy was verified in our previous study and other studies (38, 39), however, there was no significant association between blastocyst score and CM-BF aneuploidy rate in 0PN-derived blastocysts (Supplementary Table S1), which might be the result of different detection methods. Moreover, our previous study showed that the bigger pronuclear diameter (≥25 µm) of the 1PN zygote was more likely to form blastocysts (28). In this study, we measured the pronuclear diameter of the 1PN zygotes and divided them into two subgroups (<25 µm and ≥25 µm) to determine the chromosomal aneuploidy. No significant associations were observed between the pronuclear diameter, female age, polar body number, blastocyst score and aneuploidy rates of 1PN-derived blastocysts, regardless of the TE biopsy or CM-BF aneuploidy rates (Supplementary Table S2).

For the first time, our study contributes to the understanding of chromosomal aneuploidies in 0PN- and 1PN-derived blastocysts in conventional IVF cycles through TE-biopsy PGT-A and miPGT-A. Our findings indicate that the clinical application value of 0PN- and 1PN-derived blastocysts in conventional IVF should be assessed using TE-biopsy PGT-A or miPGT-A. The above results will help provide accurate genetic counseling and embryonic selection for IVF couples with 0PN or 1PN embryos. Furthermore, in terms of consistency, the miPGT-A using blastocoel fluid enriched culture medium is promising as an alternative to TE-biopsy PGT-A for aneuploidy testing of 0PN- or 1PN-derived blastocysts in conventional IVF.

## Data Availability

The datasets presented in this study can be found in online repositories. The names of the repository/repositories and accession number(s) can be found below: https://submit.ncbi.nlm.nih.gov/subs/sra/SUB10792759/overview.
