# Supplementary material for: The aneuploidy testing of blastocysts developing from 0PN and 1PN zygotes in conventional IVF through TE-biopsy PGT-A and minimally invasive PGT-A
Source: Front Reprod Health. 2022 Sep 5;4:966909. doi: 10.3389/frph.2022.966909 (PMC9580634; doi:10.3389/frph.2022.966909)
Supplement: Supplementary file 1 [file Table_1_v1.docx]

**Supplementary table 1**

Analysis between related factors and aneuploidy rates of 0PN-derived blastocysts, % (n).

|  |  | **TE-biopsies** | |  |  | **CM-BF** | |  |  |
| --- | --- | --- | --- | --- | --- | --- | --- | --- | --- |
| **Characteristic** |  | **Euploid rate** | **Aneuploid rate** | ***P* value** | **OR (95% CI)** | **Euploid rate** | **Aneuploid rate** | ***P* value** | **OR (95% CI)** |
| Female age | <35 year | 92.9% (39/42) | 7.1% (3/42) | 0.001 | 0.085 (0.019-0.375) | 66.7% (28/42) | 33.3% (14/42) | 0.510 | 1.455 (0.477-4.431) |
|  | ≥35 year | 52.6% (10/19) | 47.4% (9/19) |  |  | 57.9% (11/19) | 42.1% (8/19) |  |  |
| Polar body number | 1pb | 78.3% (36/46) | 21.7% (10/46) | 0.482 | 0.554 (0.107-2.870) | 58.7% (27/46) | 41.3% (19/46) | 0.146 | 0.355 (0.088-1.433) |
|  | 2pb | 86.7% (13/15) | 13.3% (2/15) |  |  | 80.0% (12/15) | 20.0% (3/15) |  |  |
| Blastocyst score | ≥BB | 89.2% (33/37) | 10.8% (4/37) | 0.038 | 4.125 (1.080-15.762) | 67.6% (25/37) | 32.4% (12/37) | 0.464 | 1.488 (0.513-4.313) |
|  | <BB | 66.7% (16/24) | 33.3% (8/24) |  |  | 58.3% (14/24) | 41.7% (10/24) |  |  |

PN, pronuclear; TE, trophectoderm; CM-BF, culture media with blastocoel fluid; pb, polar body.
