# Supplementary material for: The aneuploidy testing of blastocysts developing from 0PN and 1PN zygotes in conventional IVF through TE-biopsy PGT-A and minimally invasive PGT-A
Source: Front Reprod Health. 2022 Sep 5;4:966909. doi: 10.3389/frph.2022.966909 (PMC9580634; doi:10.3389/frph.2022.966909)
Supplement: Supplementary file 2 [file Table_2_v1.docx]

**Supplementary table 2**

Analysis between related factors and aneuploidy rates of 1PN-derived blastocysts, % (n).

|  |  | **TE-biopsies** | |  |  | **CM-BF** | |  |  |
| --- | --- | --- | --- | --- | --- | --- | --- | --- | --- |
| **Characteristic** |  | **Euploid rate** | **Aneuploid rate** | ***P* value** | **OR (95% CI)** | **Euploid rate** | **Aneuploid rate** | ***P* value** | **OR (95% CI)** |
| Female age | <35 year | 60.0% (12/20) | 40.0% (8/20) | 0.577 | 0.500 (0.044-5.700) | 60.0% (12/20) | 40.0% (8/20) | 0.577 | 0.500 (0.044-5.700) |
|  | ≥35 year | 75.0% (3/4) | 25.0% (1/4) |  |  | 75.0% (3/4) | 25.0% (1/4) |  |  |
| Polar body number | 1pb | 63.6% (7/11) | 36.4% (4/11) | 0.916 | 1.094 (0.208-5.756) | 45.5% (5/11) | 54.5% (6/11) | 0.121 | 0.250 (0.043-1.443) |
|  | 2pb | 61.5% (8/13) | 38.5% (5/13) |  |  | 76.9% (10/13) | 23.1% (3/13) |  |  |
| Blastocyst score | ≥BB | 76.9% (10/13) | 23.1% (3/13) | 0.121 | 4.000 (0.693-23.089) | 76.9% (10/13) | 23.1% (3/13) | 0.121 | 4.000 (0.693-23.089) |
|  | <BB | 45.5% (5/11) | 54.5% (6/11) |  |  | 45.5% (5/11) | 54.5% (6/11) |  |  |
| Pronuclear diameter | <25μm | 33.3% (2/6) | 66.7% (4/6) | 0.104 | 0.192 (0.026-1.401) | 50.0% (3/6) | 50.0% (3/6) | 0.469 | 0.500 (0.077-3.265) |
|  | ≥25μm | 72.2% (13/18) | 27.8% (5/18) |  |  | 66.7% (12/18) | 33.3% (6/18) |  |  |

PN, pronuclear; TE, trophectoderm; CM-BF, culture media with blastocoel fluid; pb, polar body.
